# Traveling for Health

**Published:** 1868-05

**Authors:** 


### Hall's Journal of Health, May, 1868.

### TRAVELING FOR HEALTH.

The number of persons going abroad for summer travel is becoming greater every year, and with the increasing thrift of the country the luxury of a European tour is placed within the power of a larger number of persons than ever before, in the history of our country. A writer in the *Home Journal*, in an interesting gossippy letter from Rome, says: "One scarcely realizes that he is not in New York; there are so many Americans there that the English language is spoken fluently on every side, and even the waiters at the table have become familiar with it." In *Harper's Handbook of Foreign Travel*, corrected every year up to March 1st, so as to give the very latest reliable information as to routes of travel, modes of communication, cost of conveyance, hotel charges and other noteworthy matters, the statement is made: "The author has seen at one time sitting in the court yard of the Hotel du Louvre, Paris, twenty-nine Americans, two Frenchmen, three Englishmen, and one Russian; he has seen, in the Mediterranean Hotel in Jerusalem, thirteen Americans, one Englishman, two Frenchmen and three Spaniards; and at Shepherd's Hotel, at Cairo, in Egypt, over one-half of the visitors were Americans."

Going abroad gives a breadth of view, opportunities of observation, and facilites of information as to countries, places, men and things, which are not to be obtained in any other way at any expense; a fund for conversation is laid in, which is exhaustless and lasts for life; and it will soon come to be that not to have been abroad will make a man considered as much behind the times as one now is who does not read a newspaper. The reader who has not been abroad is conscious of the fact that the moment he learns that the person to whom he is listening has been to Europe, there is an involuntary and instinctive increase of appreciation. And then, in traveling through our own country, and meeting with strangers, we immediately have a common fund of interesting remark and pleasurable reminiscences, as soon as it becomes known that both parties have visited the same places. On the other hand, those who have traveled have it in their power to make themselves exceedingly interesting and instructive to those of their friends who have not yet had the opportunity, and the more so to those who contemplate a visit to the other hemisphere. The traveler knows, too, that ever after he has visited a place of note abroad, his mind is instructively and pleasantly carried back to Paris or Rome or London or St. Petersburgh, whenever he sees either of these names in print, and he takes a greater and more intelligent interest in whatever may be said about them, than he could possibly do had he never visited them; and these are pleasures that cease only with life.

Again, our own country is so rapidly filling up with foreigners whose economy and industry and thrift are giving them a social and business status, which requires imperatively that we should understand and speak their language to a far greater extent than has ever been known; and a few months in a foreign city will give, especially to children, opportunities and facilities for catching the correct pronunciation and full meaning of words, than could ever be learned at home.

It has become a recognized and indisputable fact, that no person ought to teach a language not natural to him, nor can he teach correctly any other language than his own, because otherwise it is impossible to come at a correct pronunciation, or at the full and true meaning of words and phrases; and it is confessedly more difficult to unlearn a faulty pronunciation than to learn a new one; hence, those families should consider themselves greatly favored who can take their children abroad when the youngest is not over ten years of age, and who can remain several years, especially as better instructors can be had in any branch of practical or æsthetic study for thirty cents an hour than for three dollars an hour here, especially in such places as Halle, Dresden, Heidelbergh and similar localities of universities. And then, on the return home two grand practical ideas fill the whole mind: first, that ours is the most glorious country on the face of the earth; second, that our religion and our religious privileges are sweeter, dearer than honey from the honeycomb.

With these views we propose to give a few hints to our readers, hoping they may learn from our own experiences, and that they or their children may have an opportunity of putting into practice, at the cost of reading what has cost us time and money to become possessed of.

In the first place, arrange that you shall have no business anxieties to interfere with your fullest enjoyment.

Second. Take the nearest and dearest ones with you, that is, wife and children.

Third. Arrange for a prompt and plentiful supply of money in any part of the world, according to your circumstances.

Fourth. Don't presume on what you may think you are at home; if you are Corporal, Lieutenant, Captain, Major, Colonel, Brig.-Gen. Bull, put your name down and act as plain John Smith, gentleman, because you are estimated by waiters, landlords and fellow-travelers from the amount of the gentleman you exhibit; a gentleman, in traveling, never tells who he is, what he is, or what he has been, unless he is compelled; he under all circumstances acts the gentleman, and there, as far as information concerning himself is concerned, he stops.

Fifth. Bring into requisition as large an amount of patience, consideration and forbearance, as is possible for you to procure; it is worth what money can never purchase, and will save you from innumerable discomforts and annoyances; it will save you from many a humiliating self-condemnation, from many a bitter remorse, and from the contempt of many a looker-on.

Sixth. Reader, there is human nature all over the world; is not that a tremendous truth? By any thing we have said you will be able to travel through all lands in quiet, peace and a reasonable degree of comfort; but the most of us like to be noticed a little; we want to have some consideration, some attention shown to us, beyond what would be bestowed on plain Mr. John Smith, gentleman. The most that will be done for him will be to let him alone, and take care of himself the best way he can; but you, reader, would rather have some little fuss made over you; you would like to have the best room at hotels, the best berths on boats, railways and steamships, the most comfortable seats at the table, and the best pieces of turkey, roast beef and porter-house. It is pleasant, too, to have waiters bow to you profoundly, and obsequious clerks and landlords to be ever at your elbow calling out "this way if you please, sir," and opening a passage for you in every crowd.

Will money do all this? No, sir. Will politeness secure it? Not at all. Will titles ensure it? Not by any means; but there is a way by which all these attentions can be secured in whatever clime or country you may travel. Persons of all languages alike understand its power, and bow to it with instinctive cordiality, and satisfaction too, because the article pays its way; it is cashed on sight wherever the sun shines on a community of human beings. We wonder if any reader has divined this universal talisman. Take a pretty girl along; if you have not a daughter or sister, look around among your country cousins, and wherever you find her pay her expenses, and in the long run you will find it largely remunerative in the direction we have named. We have tried it and speak from experience. We once took a really beautiful girl with our family, as nurse for our youngest child, and we shall never forget the partialities shown us everywhere; the fact is, it made such an impression on our mind that we resolved that if we ever made an important journey again we would arrange in some way to have some young, beautiful face along.

Seventh. It is better and more instructive to see one place well than a dozen places imperfectly. If you have a month to spare in England or Italy, it will be more satisfactory to yourself, and far more instructive to those with whom you may converse ever after, to give that month to London or Paris or Rome, than to the whole of the countries of which they are the capitals or great centers. Definite, specific knowledge—knowledge in detail—is more valuable than what is general, and as you can enter into details you can proportionably interest and instruct those who listen to you.

*Harper's Handbook for Travelers in Europe* gives details by which you will be able to see the most interesting things in the large cities in proportion to the time you have to spare, whether for one day, one week, one month or six; for example, suppose you make your headquarters at Charing Cross or Spring Garden, both near the Palace of the Queen; then directions are given by which you can make daily excursions for several weeks, each day taking a different road, walking or riding; in this way an amount of health and bodily vigor may be secured which, of itself, would be of incalculable value to many.

Eighth. If a family from a city or large town goes abroad with a determination to make the most of their time, and see all that is possible, it can be arranged that at night they can form their plans for the morrow, take an early breakfast, trudge until noon, when dinner should be taken, which perhaps it would be better to do wherever you happen to be, to save the time of going and returning home; then trudge again until sundown, arranging never to be out later, because the body then is weary and tired, the spirits are saddened, and the circulation languid, leaving the body most susceptible to the ugly dampness of sundown, and those pernicious emanations from the soil which, in all latitudes, in warm weather, are fruitful of many diseases, troublesome, painful, dangerous, and even fatal; another reason is, strangers should arrange to to be at their own homes as soon as night comes, as a matter of personal safety; almost all the knock-downs and robberies and garottings in cities take place after nightfall; besides, it is wisdom in any traveler to get all the sleep possible at night, so that both body and mind may be fully alive to the programme of the following day. It promotes activity of body, vivacity of spirits, cheerfulness of disposition, and a general feeling of kindliness to all the world, all of which feelings are essential to the highest enjoyment of foreign travel.

A course of this kind is followed by a voracious appetite, and by a soundness of sleep, and a lithesomeness of spirits, which, for the recuperation of health, are more efficient in all ordinary ailments than all the resources of druggery. By carrying out plans of this kind firmly, day after day the year round, there would be an invigoration of the constitutions of children brought up in the luxuriousness and bodily inactivities of cities, which would be of life-long value, and which could not possibly be secured in any other way.

### EXPENSES OF FOREIGN TRAVEL.

Those who are weak-minded enough to want to "make a splurge" can spend any amount of money, and receive therefor a commensurate degree of contempt. It is generally said of Americans that they make great fools of themselves in very many cases, seeming to think that the more money they spend the happier they will be, and the more consideration will be shown them——by servants, chambermaids, bootblacks and the like; hence they affect first-class hotels, first-class seats in railway cars, and first-class everything.

But we are writing for those of moderate means and who wish to travel unobtrusively and learn a great deal. The experience of many travellers is that there is more comfort and better attention to be had at second rate hotels than at those which are termed first rate ; you travel more safely in second rate rail cars than in the first class and save a third of your money.

PASSAGE TO EUROPE.

can be had in steamships, at from eighty dollars to two hundred ; in sailing vessels for about seventy dollars in paper ; the steamship requiring ten days, the sailor twenty ; if the winds are contrary persons in steamships are liable to be very sea sick during the entire voyage ; while in a sailing vessel it usually passes off in a few days ; we advise that the ocean be crossed at least once in a sail vessel as it gives experiences, not to be had on the steamship and one can know, as they never can by mere reading what it is, to " see a ship, or ship a sea," and can more properly appreciate the descriptions of sea life which were written before the days of steam. The nearer the centre of the ship, the less seasickness will there be.

HOTELS.

Money will be saved and a great deal of trouble, when it is purposed to remain in one place beyond a day or two, to arrange before hand to have private lodgings ready for you, and by being on the alert you can bespeak places of this kind in advance, all along your journey. Private houses are less expensive than hotels ; but a cheaper plan, and one securing a larger amount of comfort and independence, and home feeling, is to rent furnished apartments ; then you can invite friends to meet you, and can make such arrangements as to eating as will suit your taste and purse. A lady friend just returned from a year or two's journey abroad, with her daughters, without any other protector than a lady-like deportment and a cultivated mind, says she procured very fine apartments in Rome, last winter ; had a person to bring her whatever was wanted to be eaten, at any hour named, and as soon as the repast was ended every thing was removed, and they were ready for a new jaunt, and all this at an expense of about a gold dollar every day for each person ; if a carriage was needed, one of the finest turnouts could be had for an afternoon, for half-a-dozen persons, for less than two dollars in gold. The same things can be had in the University towns, in Germany, especially in winter, for about seventy-five cents a day, in gold, for each person ; and the very best instructions in the classics, languages, mathematics, music, painting, and the like, at from ten to thirty cents an hour.

It is impossible to travel advantageously in Europe without a guide-book, or living guide, or "courrier," as they are called, and Harper advises to purchase guide-books, or remain at home. To make the "Tour of Europe," some twenty or thirty guide-books will be needed, costing, perhaps, a hundred dollars, with the disadvantage of being two or three, or ten years old; and then to be lugging these books around with you is inconvenient and expensive. But the traveling public are greatly indebted to Harper & Brothers, for publishing one book of seven hundred pages, with a flexibe leather cover, in pocket book form, for five dollars, and which is worth all the guide-books on the Continent, because it gives succinctly all the desirable knowledge which the others do, and brought up to the 1st day of March, of each year, by W. Pembroke Fettridge, who resides abroad for the purpose of gathering the latest information on all subjects desired by the tourist. The full title of the book is suggestive, "Harper's Hand-book for Travelers in Europe and the East, being a guide through Great Britain and Ireland, France, Belgium, Holland, Germany, Italy, Sicily, Egypt, Syria, Turkey, Greece, Switzerland, Tyrol, Spain, Russia, Denmark and Sweden, with a railroad map, corrected up to 1868, and a map embracing colored routes of travel in the above countries, seventh year, published by Harper Brothers, Franklin Square, New York City; Galignani & Co., No. 224 Rue Rivoli, Paris; and in London by Sampson-Low, Son & Co., and J. A. Adams, No. 59 Fleet street." It tells you where are the best and second best hotels, the names of the bankers in the principal cities of Europe; where you can get supplies of clothing; the value of the coins of different countries; what places ought to be visited, with a succinct history of each country, city and village, in any route; every now and then throwing in a bit of practical philosophy, of considerable value, a specimen of which is found on the first page of the Introduction. "It behooves us to travel with other stores besides our purse and passport. A man must carry knowledge with him, if he would bring knowledge home.

Everybody now has an excuse for travel; if rich, to enjoy; if poor, to retrench; if sick, to recover; if studious, to learn; if learned, to relax from study. All should remember that not the least important requisite for a traveler, is a ready stock of good temper and forbearance. Let your motto be, "keep cool." Good humor will procure more comforts than gold. If you think you are imposed upon, be firm; custom has established certain rules and charges, and any deviation from them is soon detected, and, unless unnecessary trouble has been given, firmness and good temper will serve you better and more readily than violence.

"We, as a nation, have, unfortunately, acquired a reputation abroad of great prodigality in our expenditures, and in the East we are charged twenty per cent. more than any other nation for what we purchase; still, it is an unhappy feeling to think that we must always be on our guard. Many set out with that deeply to be regretted impression, and are rendered miserable by imagining they are the victims of imposition wherever they go, and by degrees become despicably mean, and grumble at every charge they do not understand. Tristam Shandy's reflections on this subject are worth quoting, "yet, notwithstanding all this, and a pistol tinder-box which was filched from me, at Sienne, and twice that I paid five pauls (fifty cents) for two half-boiled eggs, I do not think a journey through France, or Italy, so bad a thing as some people would make you believe, provided a man can keep his temper all the way.'

"Wherever you are, it is best for you to fall into the manners and customs of the place; it may be inconvenient, but it is less so than in running counter to them."

From the above extracts, the reader will see the importance of supplying himself with "Harper's Hand-book of Foreign Travel," before he leaves New York, and then he will have the advantage of being able to study it during the passage.

### MONEY MATTERS.

Bring all the money you can to New York, and deposite it in some banking house, and for it they will give you what is called "a letter of credit" for that amount, which letter you sign, it giving a description of your person, age, stature, forehead, eyes, nose, mouth, chin, hair, complexion, face; they then send circulars to every principal place to which you purpose going, to their branch house; when you arrive at the place, if you want a supply of money, all that you have to do is to carry your letter with you, and on presentation the amount is paid and named in your letter, to which you append your signature as a receipt, and thus each succeeding banking-house knows how much has been paid on your New York deposite, and you can't overdraw; on the other hand, if you loose your letter, it will not benefit the finder, as he would not answer to your description nor be able to imitate your signature; and even if he did, and the money was paid him, it would not be your loss, because it is their business to know you and to pay it to you, and to no one else; if lost, you can have your letter renewed by applying to the United States Consul, or Minister, at that place, and making the matter known at the banking house. It is better not to draw much at a time, so as to avoid much loss if mislaid, and to offer no special inducement to robbers.

Let your baggage be as compact as possible; carry no sealed letters with you, and avoid taking commissions from friends; carry nothing with you that is contraband, nothing that it is necessary to conceal; so that when custom house officers ask to search your trunks, you can hand them the keys with perfect indifference; this will disarm them of suspicion, and cause them to make a speedy and careless inspection. It is their sworn duty to make such examination, and it is your duty to afford them promptly, willingly, and courteously, every facility for discharging their duty.

There are a number of little observances in travel, if attended to, will save a world of trouble every day, almost.

When you are at a hotel, or other stopping place over a day, call for your bill and settle it the night before starting; it saves time, prevents hurry, mistakes, and often angry words, and irritating misunderstandings. Not a less comfort is it to arrange to be at your starting place a full quarter of an hour before the time, and you will be more than paid for your trouble, in the opportunity of quietly observing the ridiculous positions in which many place themselves by their necessity of haste. Half a minute too late, a quarter of a minute too late ; what a predicament, equal sometimes to the loss of a day, a week, a month.

### TO ENJOY TRAVEL,

A person must be well, and a few observations on this subject are in point.

Eat at regular hours, thrice a day, and not between times. Dress abundantly warm, sleep at night, travel in the day time.

Let nothing interfere with the prompt and regular performance of all the bodily functions. One thing a traveler must always bear in mind, never part with your letter of credit or your passport, never let them go out of your sight. Write for your passport at least two weeks before you come to New York, so that it may be ready on your arrival. Address A. C. Wilmarth, United States Passport Agent, No. 41 Chambers street, New York, and send him ten dollars, five of which he must pay to the United States, and the remainder is for his services, and for having it properly mounted on linen. bound in morocco cases, with extra leaves to receive the visés, when the passport proper is full, and have your name distinctly lettered in gold on the cover.

There is a thoughtless and unjust mistake which nearly all new travelers make, generalizing too quickly and drawing conclusions therefrom, especially in an unfavorable direction. If such an one is imposed upon by a hotel keeper, by a merchant, by a hackman, by a railroad employeé, by a police officer or consul, he immediately estimates the character of the whole class, if not the whole nation, from that of the individual ; it requires but a thought to see what gross injustice would be done to New York, as a city, or our whole nation, if persons who flit through our country by rail, at the rate of forty miles an hour, should form an opinion of the character of our people from the drinking, the oaths, the trickeries and the vulgarities which came under his notice on the great lines of travel, in hotels, at steamboat landings, railroad stations, etc. The true character of a people can only be ascertained by remaining sometime at a place and by having some little opportunity of social intercourse.

There is so much to see in Italy, and so much to be enjoyed in contemplating localities, famous for two thousand years, that whoever contemplates going abroad, should take this interesting country in his route ; but his journeyings there and the information derived from them, will be made doubly instructive and enjoyable by reading "Venetian Life," and "Italian Journeyings," by W. D. Howells, just published by Hurd & Houghton, 459 Broome street, New York ; to us these books have been irresistably amusing ; there is scarcely a page that does not bring a smile or a loud guffaw at the dry humor of the talented writer, while his insight of human nature, and Italian human nature especially, seems to be intuitive. He is very outspoken, and does not conceal his prejudices and dislikes ; there is a piquancy and an instructiveness in his narrations, which enchains the reader's attention most delightfully. Here is a specimen or two of his putting things : "Like the Englishman who had no prejudices, I do hate a Frenchman, and there were many of them aboard, in whose company I could hardly have been happy, had I not seen them horribly sea-sick. I have seen commercial travelers of all nations, and I think I must award the French the discredit of producing the most odious commercial travelers in the world. The Englishman of this species, wraps himself in his rugs, and rolls into his corner defiantly, but not aggressively boorish ; the Italian is almost a gentleman ; the German is apt to take sausage out of a newspaper and eat it with his penknife ; the Frenchman aggravates human nature beyond endurance by his restless ill-breeding, and his evident intention not only to keep not only all his own advantages, but to steal some of yours upon the first occasion." Should the reader contemplate going abroad this summer, and by way of variety take Greenland in his way, he will derive important information before hand by reading the "Open Polar Sea," by Dr. Hays, issued in fine style by this same enterprising house ; and the cost of the volume will never be regretted. Since writing the above of Mr. Howels' volumes, we are gratified to find that they are pleasingly commended by others ; " Harper's Monthly " says : " The most vivid, accurate and poetic description of Venice that we recal," while the "North American Review" observes, "We know of no single word which will so fitly characterize Mr. Howells' new volume about Venice, as "delightful." The stately " Westminster Review " says : " There is hardly a feature of Venetian life that escapes his sympathetic observation." The "London Contemporary Review" declares that " Every sentence of this charming book is characteristic ; it is the very model of what a light book of travels ought to be."

